# Late-onset Huntington’s disease with 40–42 CAG expansion

**DOI:** 10.1007/s10072-019-04177-8

**Published:** 2019-12-09

**Authors:** Elisa Capiluppi, Luca Romano, Paola Rebora, Lorenzo Nanetti, Anna Castaldo, Cinzia Gellera, Caterina Mariotti, Antonella Macerollo, M. Giuliana Cislaghi

**Affiliations:** 1 Department of Neurology, ASST Valle Olona, Saronno, Italy; 2 Department of Clinical Sciences “Luigi Sacco”- L. Sacco Hospital, University of Milan, Milan, Italy; 3 Medical Statistics School, University of Milano-Bicocca, Milan, Italy; 4 Unit of Medical Genetics and Neurogenetics, Fondazione IRCCS Istituto Neurologico Carlo Besta, Milan, Italy; 5 Department of Neurology, The Walton Centre NHS Foundation Trust, Liverpool, UK; 6 School of Psychology, Faculty of Health and Life Sciences, University of Liverpool, Liverpool, UK

**Keywords:** Huntington’s disease, Epidemiology, Age of onset

## Abstract

**Introduction:**

Huntington’s disease (HD) is a rare autosomal dominant neurodegenerative disorder caused by a CAG expansion greater than 35 in the *IT-15* gene. There is an inverse correlation between the number of pathological CAG and the age of onset. However, CAG repeats between 40 and 42 showed a wider onset variation. We aimed to investigate potential clinical differences between patients with age at onset ≥ 60 years (late onset-HD) and patients with age at onset between 30 and 59 years (common-onset HD) in a cohort of patients with the same CAG expansions (40–42).

**Methods:**

A retrospective analysis of 66 HD patients with 40–41–42 CAG expansion was performed. Patients were investigated with the Unified Huntington’s Disease Rating Scale (subitems I–II–III and Total Functional Capacity, Functional Assessment and Stage of Disease). Data were analysed using *χ*^2^, Fisher’s test, *t* test and Pearson’s correlation coefficient. GENMOD analysis and Kaplan-Meier analysis were used to study the disease progression.

**Results:**

The age of onset ranged from 39 to 59 years in the CO subgroup, whereas the LO subgroup showed an age of onset from 60 to 73 years. No family history was reported in 31% of the late-onset in comparison with 20% in common-onset HD (*p* = 0.04). No difference emerged in symptoms of onset, in clinical manifestations and in progression of disease between the two groups.

**Conclusion:**

There were no clinical differences between CO and LO subgroups with 40–42 CAG expansion. There is a need of further studies on environmental as well genetic variables modifying the age at onset.

**Electronic supplementary material:**

The online version of this article (10.1007/s10072-019-04177-8) contains supplementary material, which is available to authorized users.

## Introduction

Huntington’s disease (HD) is a rare autosomal dominant neurodegenerative disorder caused by an expansion of cytosine-adenine-guanine (CAG) repeats (≥ 36) in the exon 1 of the Huntingtin (HTT) gene, encoding for a stretch of polyglutamine (polyQ) in the Huntingtin protein [22]. This gene is polymorphic and unstable; ‘*intermediate alleles*’ contain 27–35 CAG repeats showing a significant degree of instability and a propensity for expansion during spermatogenesis compared with oogenesis [23]. In other words, a healthy male with the intermediate allele has a higher probability of producing an offspring with the HD allele containing ≥ 36 CAG repeats. Carrier with 36 to 39 CAG repeats may or may not develop the signs and symptoms of Huntington’s disease in lifespan (‘*reduced penetrance alleles*’), while people with 40 or more repeats always develop the disorder [22].

HD prevalence in the Western hemisphere is 7–10/100,000 [10, 19]. The duration of the disease varies considerably with an average of approximately 19 years [19].

The age at onset in HD is the time when a carrier of the mutated gene develops unequivocal HD signs, particularly motor signs [21]. However, it is very well known that psychiatric and cognitive symptoms might onset earlier than motor signs [19].

The age of onset is inversely correlated with the CAG expansion. The peak of incidence is in the fourth-fifth decade (common-onset, CO), being clinically characterized by movement disorders (chorea, dystonia, gait and balance impairment, ataxia, dysarthria, dysphagia, oculomotor dysfunction), behavioural abnormalities (depression, anxiety, mania, obsessive-compulsive disorder, impulsive disorder, suicidal thoughts, paranoid delusions) and cognitive decline (deficit of executive functions as attention, shifting, abstraction, impairment of verbal fluency and insight of illness).

Larger CAG expansions (> 60 CAG repeats) in the Huntingtin gene are associated with juvenile HD being symptomatic at age 20 years or younger. This is a rapidly progressive phenotype characterized by rigidity, dystonia, parkinsonism, gait disturbance, loss of hand dexterity, seizure, myoclonus, learning difficulties and dramatic prognosis particularly in those with highly expanded mutations (CAG > 80). This rare variant of HD is characterized by a shorter survival variance and accounts for only about 4–10% of all cases [8]. Langbehn et al. predicted that more than 90% of individuals with repeat sizes of ≥ 44 would present HD signs before the age of 60 [14]. However, a fair number of patients present symptoms beyond the fifth decade of life. This subgroup, named late-onset HD (LO-HD), was defined with an onset after 49 years [7, 9, 18] or in more recent studies after 59 years [4, 11, 12, 15]. LO-HD accounts between 4.4 and 25% in small cohorts [4, 7, 9, 11, 12, 15, 18] and 11.4% in a larger cohort [20]. LO-HD patients have frequently negative family history; consequentially, the diagnosis might be more difficult and the prevalence results underestimated.

LO-HD has been previously described as a phenotype characterized mainly by cognitive decline than motor symptoms. However, recent studies focused on the comparison between LO-HD and CO-HD did not confirm this view [12, 20]. In these recent studies, LO-HD patients presented more frequently motor symptoms as gait and balance impairment in addition to chorea than behavioural disorders at onset compared to the CO-HD subgroup [12, 20].

The CAG repeat size in the affected allele is lower in LO-HD compared to the CO-HD, and the number of patients with a CAG repeat in the reduced penetrance range (36–39) is significantly higher in the LO-HD compared to CO-HD [20].

Overall, CAG repeat number is linked to the 66–72% of age onset variability [16, 29]. However, the 40–50 CAG repeats determine 44% of age onset. The CAG 40–42 repeats range is the one with the higher variability in the age of onset [29].

Here, we analysed a genetically homogenous HD cohort with complete penetrance repeat expansion (> 40), in the range with higher onset variability (40–42). Moreover, we compared the clinical features of two subgroups of patients: CO-HD and LO-HD.

## Methods

Sixty-six patients with a number of CAG repeats between 40 and 42 were recruited between 2004 and 2015 at the “Luigi Sacco” Hospital (Milan, Italy) as well as at “Fondazione IRCCS Istituto Neurologico Carlo Besta” (Milan, Italy). Participants had a confirmed genetic diagnosis of HD according to standardized parameters on statistical specificity and sensitivity [13, 25, 26]. The disease onset was defined as the age of onset of motor signs according to literature data [27]. The study was approved by the ethical committees of both medical institutions. Written consent form was obtained from each participant.

Patients were divided according to age at onset: CO subgroup (37 patients) with age of onset at ≤ 59 years (range 39–59) and LO subgroup (29 patients) with onset at ≥ 60 years (range 60–73).

Patients were further analysed based on onset symptoms as following: only motor symptoms [M], motor and psychiatric symptoms (M/P), motor and cognitive symptoms (M/C) and motor, psychiatric and cognitive symptoms (M/C/P) [M+].

Participants were assessed at the time of diagnosis (T0) as well as at follow-up (FU, T1) by the Unified Huntington’s Disease Rating Scale (UHDRS, subitems I–II–III) [28], the Functional Assessment (FA) and Total Functional Capacity Scale (TFC) [24]. Stage of disease and progression index was calculated as loss of TFC units for years following Shoulson and Fahn’s study [24]. The average follow-up time was 3.5 years (range 1–11 years).

The duration of symptoms at time of diagnosis (T0) was 4 years (range 1–11 years) for all samples, 3.3 years for patient with onset ≥ 60 years (range 1–11 years) and 4.0 years for patient with onset ≤ 59 (range 1–11 years).

At time of the diagnosis, the subitem II (behavioural) of UHDRS was not administrable in seven patients CO-HD and in two patients LO-HD. The subitem III (cognitive) of UHDRS was not administrable in 18 CO-HD and 16 LO-HD. Six patients in group 1 and eight in group 2 were lost at follow-up.

## Statistical analysis

Data were described as numbers (percentages), median and quartiles. The baseline characteristics between the two groups were compared by the *χ*^2^ test (Fisher’s test was used when the expected count in any cell was lower than 5). In the two samples, Wilcoxon’s test was used to compare the clinical scores among the two groups at baseline and follow-up.

A linear regression model on the change in motor score diagnosis during follow-up (T1-T0) was also applied to assess whether the two groups showed a different profile in time accounting for the time of follow-up. The age at onset (LO-HD subgroup vs CO-HD subgroup) and follow-up time were included in the model with the latter one centred on 3 years of follow-up, in order to interpret the change after 3 years. In order to assess disease progression among the two groups, a worsening condition was defined as any increment in stage during follow-up and the probability of worsening was estimated by the Kaplan-Meier estimator. The difference among the two groups was assessed by a log-rank test. Type I error was set at 0.05.

## Results

### Demographic and genetic data

Sixty-six Caucasian patients (31 males, 35 females) were recruited in the study. Seventeen cases had 40 CAG repeats, 20 patients had 41 CAG repeats and 29 patients showed 42 CAG repeats in the IT-15 gene

Thirteen subjects (19.7%, 4 CO-HD, 9 LO-HD) had no family history for HD (Table 1). A significant difference was observed in absence of family history in LO group as compared with others (31% vs 19.7% *p: 0.0403*), while no differences were observed in type of transmission (maternal or paternal, *p* = 0.6881 (Table 1).

**Table 1:** Demographic, clinical and genetic characteristics of the studied patients’ groups: onset ≤ 59 years and onset ≥ 60

	Age onset				*χ*^2^ test
	≤ 59 (*n* = 37)		≥ 60 (*n* = 29)		*p* value
	Number	Percent	Number	Percent	
Sex					0.1927
Female	17	45.9	18	62.1	
Male	20	54.1	11	37.9	
Family history					*0.0403*
No	4	19.7	9	31	
Yes	33	80.3	20	69	
Type transmission					0.6881
Maternal	13	35.1	9	31	
Paternal	20	47	11	37.9	
CAG up					*0.0403*
40	6	16.2	11	37.9	
41	10	27	10	34.5	
42	21	56.8	8	27.6	
Onset symptom					0.4601
M	25	67.6	22	75.9	
M+	12	32.4	7	24.1	
Stage of disease at diagnosis					0.2667
1–2	32	86.5	22	75.9	
3–4	5	13.5	7	24.1	

*N* number of patients, *%* perceptual of patients, *M* only motor symptoms at onset, *M+* motor and other symptoms at onset

**Fig. 1:**
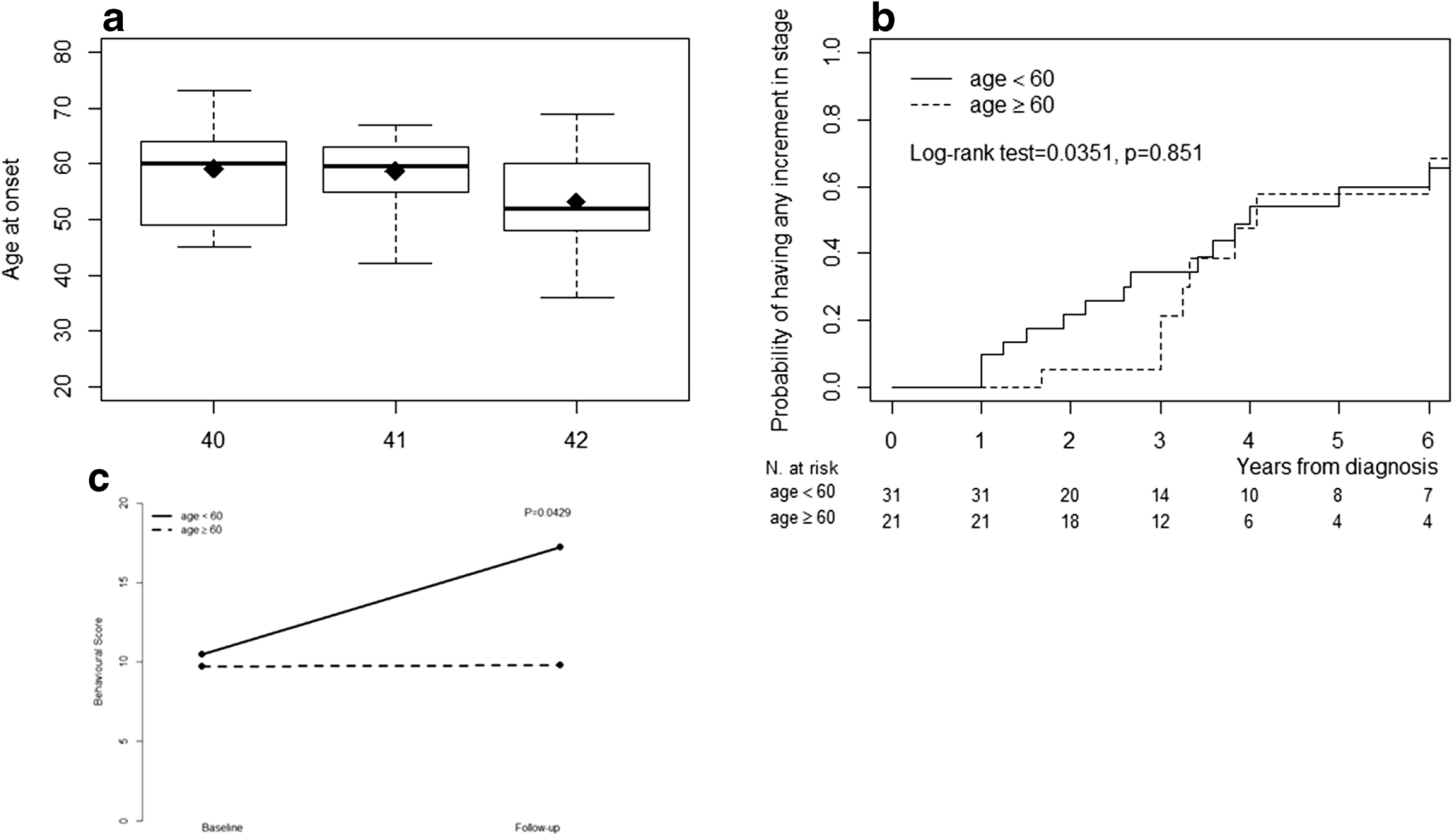
**a** The box-plot analysis showed a negative correlation between CAG repeat size and age at onset in HD patients (*p* = 0.0403), although the sample had previously been selected for CAG expansion range between 40 and 42. **b** Kaplan-Meier analysis showed the effect of age at onset on progression time to the severe stage assessed by the Total Functional Capacity Scale (log-rak test = 0.0351, *p* = 0.851). There was no difference in the prognosis between the two groups. **c** Mean behavioural score for the two groups at baseline and follow-up. *P* value refers to the difference among the two groups on the change in the behavioural score during follow-up by the linear model

The average number of CAG repeats was 40.9 ± 0.8 in the LO-HD group and 41.4 ± 0.8 in the CO-HD. A significant negative association between number of CAG repeats and age of onset was observed (*p* = 0.0403), although the sample had previously been selected for CAG expansions between 40 and 42 (Fig. 1a).

### Clinical features at onset

Twenty-five CO-HD (67.6%) and 22 LO-HD (75.9%) presented only motor symptoms (M) at onset. Twelve CO-HD (32.4%) and seven LO-HD (24.1%) had motor symptoms associated or preceded by other clinical manifestations at onset (M+), without any statistical difference among the two groups (*p* = 0.4601). In particular, eight CO-HD and five LO-HD patients had psychiatric disorders (M/P), one CO-HD and two LO-HD showed cognitive decline (M/C) and three CO-HD but any LO-HD had cognitive as well as psychiatric symptoms (M/C/P) at the time of diagnosis.

We were focused in investigating a potential profile of onset of psychiatric features compared to the movement disorder. Thus, the interval of time between the onset of each type of symptoms was investigated in the group of patients with combined phenotype (motor + psychiatric features, Table 2).

**Table 2:** Presence of cognitive and psychiatric symptoms at onset in the two patients’ group

	All		Age onset				*χ*^*2*^ test
			≤ 59		≥ 60		(Fisher *)
	Number	Percent	Number	Percent	Number	Percent	*P* value
Cognitive impairment							
No	60	90.9	33	89.2	27	93.1	0.6875*
Yes	6	9.1	4	10.8	2	6.9	
Psychiatric disorder							
No	50	75.8	26	70.3	24	82.8	0.2400
Yes	16	24.2	11	29.7	5	17.2	
Cognitive impairment + psychiatric disorder							
No	63	95.5	34	91.9	29	100	0.2497*
Yes	3	4.5	3	8.1	.	.	

*N* number of patients *%* perceptual of patients

In particular, the onset of a depressive disorder prior or associated to the onset of motor symptoms was analysed in our cohort of patients. It was found that 16 patients in total (24.3%), 11 CO-HD and 5 LO-HD, had depressive symptoms earlier or associated to the onset of abnormal movements (Table S1).

### Disease progression

Clinical characteristics of the studied patients’ groups at the baseline (T0) and at follow-up (T1) are shown in Tables 2 and 3. The years of disease at time of diagnosis (T0) was 4 years (range 1–11 years) for all samples, 3.3 years for patient with onset ≥ 60 years (range 1–11 years) and for patient with onset ≤ 59 was 4.0 years (range 1–11 years).

**Table 3:** Clinical characteristics of the studied patients’ groups (onset ≤ 59 years and onset ≥ 60) at the baseline (T0)

	Age onset				
	< 60		≥ 60		Test
	Number	Median(q1-q3)	Number	Median(q1-q3)	*P* value
Dystonia Score	37	1(0–2)	29	0(0–2)	0.4732
Chorea Score	37	7(4–8)	29	8(3–12)	0.2368
Total Motor Score	37	20(14–40)	29	26(14–43)	0.3792
Functional Assessment	37	23(16–25)	29	19(13–25)	0.1987
Total Function Capacity	37	12(9–13)	29	11(8–13)	0.2172
Behavioural Score	32	9(4–16)	27	9(2–19)	1
Symbol Digit Span	19	20(8–31)	12	15(6–24.5)	0.3174
Verbal Fluence	19	13(5–30)	13	19(9–25)	0.6618
Semantic Fluence	18	11.5(7–15)	11	7(5–16)	0.6235
Stroop Test Interference	19	20(13–40)	13	21(7–32)	0.4707
Stroop Test Verbal	19	60(45–90)	13	65(41–70)	0.7319
Stroop Test Colour	19	36(32–60)	13	52(33–66)	0.6347

*N* number of patients, *q1* first quartile, *q3* third quartile

Among the CO-HD subgroup, 86.5% had an early stage of the disease (1–2) and 13.5% had an advanced disease stage (3–4). In LO-HD subgroup, 75.9% had stage 1–2 and 24.1% had an advanced stage. The *χ*^2^ test did not show significant differences (Table 1).

The median time to the follow-up visit (T1) was 2.9 years (first–third quartile 1.8–4 years) for the 52 patients with a follow-up visit (Table 4).

**Table 4:** Clinical characteristics of the studied patients’ groups (onset ≤ 59 years and onset ≥ 60) at the follow-up (T1)

	Age at onset				Test
	< 60		≥ 60		
	Number	Median(q1-q3)	Number	Median(q1-q3)	*P* value
Dystonia Score	31	2(0–6)	21	2(0–3)	0.3471
Chorea Score	31	7(3–9)	21	7(4–12)	0.5513
Total Motor Score	31	34(23–57)	21	53(29–61)	0.2844
Functional Assessment	31	15(11–19)	21	10(6–21)	0.2365
Total Function Capacity	31	7(4–11)	21	5(3–12)	0.2613
Behavioural Score	31	15(7–28)	20	12(2.5–19)	0.0753
Symbol Digit Span	28	15.5(5–25)	12	4.5(0–16)	0.0754
Verbal Fluence	27	13(6–25)	13	8(3–17)	0.2319
Semantic Fluence	28	7.5(5–10.5)	13	7(5–9)	0.4773
Stroop Test Interference	27	17(11–24)	12	10(0.5–24.5)	0.2533
Stroop Test Verbal	27	52(35–71)	12	40(16–52)	0.1321
Stroop Test Colour	27	35(26–50)	12	26(11.5–40.5)	0.2194

*N* number of patients, *q1* first quartile, *q3* third quartile

The progression of motor score impairment per year was 3.83 (95%CI: 2.53; 5.14; *n* = 52; Fig. 1b), whereas the progression of the behavioural score per year was 1.33 (95%CI: − 0.24; 2.91; *n* = 45; Fig. 1c). The progression of disease was calculated as a variation in the clinical scores at 3-year follow-up. No significant differences were found in the two subgroups (Fig. 1b, Table S2).

Moreover, the incidence of disease progression as measured as a worsening in the TFC scale did not show significant differences among the two groups (*p* = 0.851, Fig. 1b).

## Discussion

Since the discovery of *IT-15* gene, the clinical phenotype and disease progression of LO-HD have not been clarified.

The definition of LO-HD itself is unclear. Indeed, some authors defined the late onset as ≥ 50 years [5, 7, 9, 16] while recent studies identified the LO-HD as ≥ 60 years [4, 11, 12, 15].

The number of subjects with onset in the range 51–59 years old was only 29 in this study, analysing a sample of patients with 40–42 CAG repeats in the major allele with complete penetrance. Although we have considered ≥ 60 years the age of onset of LO-HD, it would be useful to simplify the ranges of age of onset in future studies. Therefore, we propose the following ranges: (1) 0–29 years = juvenile onset, (2) 30–49 years = common onset (CO-HD) and (3) ≥ 50 years = late onset (LO-HD).

The cause underlying the variability in the age of onset with the same CAG expansion has not yet been clarified. Indeed, we found that the onset was 45–73 years in cases with 40 CAG repeats, 42–67 years in the group with 41 CAG repeats and 39–69 years in patients with 42 CAG repeats.

Our study confirmed the lack of familiarity in several cases and the non-prevalence of maternal transmission in LO patients [13, 20]. We observed a significant negative association between the number of CAG repeats and age of onset, although the sample was selected for CAG expansions between 40 and 42.

These results confirmed the role of genetic characteristics in the clinical features of the disease. However, this study highlighted the current need to investigate other factors (environmental, familial), which might play a role in the phenotype.

Some studies described the LO-HD phenotype as relatively benign with less severe features and a slower progression than CO-HD [1, 2, 6, 11, 13, 15, 17].

However, most studies enrolled patients carrying *reduced penetrance alleles* ranging from 36 to 39 CAG. However, these patients’ phenotypes have to be evaluated with caution, as these patients might not manifest HD in their lifespan.

Koutsis et al. highlighted the absence of clinical differences at onset between patients presenting before and after 60 years, but unexpectedly, a faster progression of the disease was observed in the late-onset group compared the Co-HD cases [12].

On the contrary, Chaganti et al. found a significant correlation between age of onset and CAG expansion size as well as variability in the phenotype on the base of the age of onset [3].

OOsterloo et al. showed that LO-HD patients had more frequently gait and balance impairment as first symptom, but disease progression was not milder compared to common-onset HD patients apart from motor progression [20].

We did not find significant differences in clinical features at onset between the two groups, especially regarding presence and severity of choreic movements. However, further studies are needed to assess subtle cognitive decline and motor symptoms other than chorea, such as gait abnormalities or falls in LO-HD.

Moreover, no statistical differences emerged regarding the severity of the stage of illness or the years of illness at diagnosis. Nevertheless, our impression was that LO-HD had a later diagnosis than CO-HD; in effect, the decline in cognitive and motor skills might be interpreted by ageing with unawareness of disease [11].

At the time of diagnosis, UHDRS scores did not show significant differences between the mean values in the two sub-groups, even corrected by the number of years of illness confirming the results of a recent study [12]. A further analysis was performed using a logistic regression procedure, correcting data for years of illness, genetic expansion and education only for cognitive items. Again, no significant differences were highlighted. Equally, no significant differences emerged at the averaged follow-up time.

We did not find differences in the estimated disease progression between the two sub-groups. A Kaplan-Meier analysis for the two sub-groups was obtained for the disease stage, considering the annual probability of achieving a worse disease stage; the two sub-groups did not differ significantly.

We are aware of some limitations of our study, especially, the presence of missing data. Indeed, at time of the diagnosis, the subitem II (behavioural) of UHDRS was not administrable in seven patients CO-HD and in two patients LO-HD. The subitem III (cognitive) of UHDRS was not administrable in 18 CO-HD and 16 LO-HD. Six patients in group 1 and eight in group 2 were lost at follow-up. Unfortunately, the clinical status of some patients and/or the lack of a caregiver in some cases did not make possible the administration of subitems II and III of UHDRS. Moreover, the subitem III requires a significant compliance from the patient, which was not present in some cases. The participants lost at follow-up are patients that did not attend our clinics anymore.

In addition, this study has limitations of a retrospective study. We are aware of the need of a longitudinal prospective study.

In conclusion, our study showed that there were no clinical differences between LO-HD and CO-HD for the same range of CAG expansion (40–42). Moreover, it was not possible to identify a phenotypic variant of LO-HD. Especially, the LO-HD patients might have a different pattern of symptoms at onset or a different prognosis.

Further studies assessing age at onset modifying factors, genetic, epigenetic and environmental factors are needed. Indeed, knowing the mechanisms that might delay the age of onset will help to identify new therapeutic targets, in addition to gene silencing.

## Electronic supplementary material

ESM 1 (DOCX 25 kb)
