# Supplementary material for: Late-onset Huntington’s disease with 40–42 CAG expansion
Source: Neurol Sci. 2019 Dec 9;41(4):869–76. doi: 10.1007/s10072-019-04177-8 (PMC7160095; doi:10.1007/s10072-019-04177-8)
Supplement: Supplementary file 1 — (DOCX 25 kb) [file 10072_2019_4177_MOESM1_ESM.docx]

**Table S1:** Clinical phenotypes at the disease onset

**Motor + Cognitive onset**

| AO | famil | Trasm | CAG | O Symp | Depr | AO-C | AO-D | AO-B | age diag | latency |
| --- | --- | --- | --- | --- | --- | --- | --- | --- | --- | --- |
| 52 | no |  | 41 | M+C | 1 | 52 | 52 |  | 53 | 0 |
| 67 | no |  | 42 | M+C | 0 | 67 |  |  | 69 | 0 |
| 73 | no |  | 40 | M+C | 1 | 73 | 68 | 74 | 75 | 0 |

**Motor + Cognitive + Psychiatric Onset**

| AO | famil | Trasm | CAG | O Symp | Depr | AO-C | AO-D | AO-B | age diag | latency |
| --- | --- | --- | --- | --- | --- | --- | --- | --- | --- | --- |
| 44 | si | M | 42 | M+P+C | 0 | 44 |  | 41 | 47 | 3 |
| 52 | si | M | 42 | M+P+C | 0 | 48 |  | 44 | 52 | 4 |
| 56 | si | M | 42 | M+P+C | 1 | 52 | 52 | 52 | 56 | 4 |

**Motor + Psychiatric Onset**

| AO | famil | Trasm | CAG | O symp | Depr | AO-C | AO-D | AO-P | age dia | latenza |
| --- | --- | --- | --- | --- | --- | --- | --- | --- | --- | --- |
| 42 | no |  | 41 | M+P | 0 |  |  | 32 | 42 | 10 |
| 45 | si | M | 40 | M+P | 1 |  | 45 | 45 | 45 | 0 |
| 52 | si | P | 42 | M+P | 1 |  | 52 | 52 | 52 | 0 |
| 54 | si | P | 42 | M+P | 0 |  |  | 54 | 54 | 0 |
| 55 | si | P | 41 | M+P | 0 |  |  | 55 | 56 | 0 |
| 58 | si | M | 42 | M+P | 0 |  |  | 58 | 59 | 0 |
| 59 | si | P | 41 | M+P | 0 |  |  | 55 | 63 | 4 |
| 59 | si | M | 40 | M+P | 0 |  |  | 53 | 60 | 6 |
| 60 | si | P | 40 | M+P | 0 |  |  | 60 | 61 | 0 |
| 62 | si | M | 42 | M+P | 0 |  |  | 62 | 63 | 0 |
| 63 | no |  | 41 | M+P | 0 | 67 |  | 60 | 74 | 3 |
| 64 | no |  | 40 | M+P | 0 |  |  | 64 | 64 | 0 |
| 66 | si | P | 41 | M+P | 0 | 75 |  | 65 | 74 | 1 |

**Depressive symptoms at the onset**

| AO | famil | Trasm | CAG | O Symp | Depr | AO-C | AO-D | AO-P | age dia | latenza |
| --- | --- | --- | --- | --- | --- | --- | --- | --- | --- | --- |
| 45 | si | M | 40 | M+P | 1 |  | 45 | 45 | 45 | 0 |
| 47 | si | M | 42 | M | 1 |  | 46 |  | 47 | 1 |
| 48 | si | P | 40 | M | 1 | 56 | 35 | 53 | 59 | 13 |
| 49 | si | P | 42 | M | 1 |  | 46 |  | 49 | 0 |
| 49 | si | P | 40 | M | 1 | 50 | 49 | 52 | 50 | 0 |
| 50 | si | P | 42 | M | 1 |  | 42 |  | 53 | 8 |
| 52 | si | P | 42 | M+P | 1 |  | 52 | 52 | 52 | 0 |
| 52 | no |  | 41 | M+C | 1 | 52 | 52 |  | 53 | 0 |
| 56 | si | M | 41 | M | 1 |  | 55 |  | 60 | 1 |
| 56 | si | M | 42 | M+P+C | 1 | 52 | 52 | 52 | 56 | 4 |
| 58 | si | P | 42 | M | 1 | 59 | 51 | 59 | 60 | 7 |
| 60 | no |  | 42 | M | 1 | 69 | 60 | 68 | 69 | 0 |
| 62 | si | M | 40 | M | 1 |  | 38 | 66 | 67 | 24 |
| 65 | si | P | 42 | M | 1 |  | 61 |  | 68 | 4 |
| 65 | si | P | 41 | M | 1 | 68 | 52 | 68 | 66 | 7 |
| 73 | no |  | 40 | M+C | 1 | 73 | 68 | 74 | 75 | 5 |

**Table S2:** Difference on the variation/change in the clinical score during follow-up (T1-T0) among the two groups (group 2 [LO-HD] versus group 1 [CO-HD]). Results from the linear model on the change in the clinical score during follow-up (dependent variable) by time of follow-up, centered at 3 years, and group (independent variables).

|  | N | Difference (95%CI) | P |
| --- | --- | --- | --- |
| Dystonia Score | 52 | -0.8(-2.3;0.8) | 0.3483 |
| Chorea Score | 52 | -0.1(-3.1;2.9) | 0.9250 |
| Total Motor Score | 52 | 5.3(-2.9;13.5) | 0.2038 |
| Functional Assessment | 52 | -1.6(-4.3;1.1) | 0.2453 |
| Total Function Capacity | 52 | -0.05(-1.7;1.6) | 0.9551 |
| Behavioural Score | 45 | -6.9(-13.6;-0.2) | 0.0429 |
| Symbol Digit Span | 18 | -6.8(-18.3;4.7) | 0.2462 |
| Verbal Fluence | 19 | -6.3(-12.2;-0.5) | 0.0349 |
| Semantic Fluence | 20 | -4.7(-8.8;-0.5) | 0.0281 |
| Stroop Test Interference | 19 | 6.2( -3.7;16.0) | 0.2198 |
| Stroop Test Verbal | 19 | 3.7(-15.9;23.3) | 0.7135 |
| Stroop Test Colour | 19 | -0.9(-17.9;16.2) | 0.9191 |
